# Nobiletin inhibits human osteosarcoma cells metastasis by blocking ERK and JNK-mediated MMPs expression

**DOI:** 10.18632/oncotarget.9106

**Published:** 2016-04-29

**Authors:** Hsin-Lin Cheng, Ming-Ju Hsieh, Jia-Sin Yang, Chiao-Wen Lin, Ko-Haung Lue, Ko-Hsiu Lu, Shun-Fa Yang

**Affiliations:** ^1^ Institute of Medicine, Chung Shan Medical University, Taichung 40201, Taiwan; ^2^ Cancer Research Center, Changhua Christian Hospital, Changhua 500, Taiwan; ^3^ Department of Medical Research, Chung Shan Medical University Hospital, Taichung 40201, Taiwan; ^4^ Institute of Oral Sciences, Chung Shan Medical University, Taichung 40201, Taiwan; ^5^ Department of Dentistry, Chung Shan Medical University Hospital, Taichung 40201, Taiwan; ^6^ School of Medicine, Chung Shan Medical University, Taichung 40201, Taiwan; ^7^ Department of Pediatrics, Chung Shan Medical University Hospital, Taichung, Taiwan; ^8^ Department of Orthopedics, Chung Shan Medical University Hospital, Taichung 40201, Taiwan

**Keywords:** nobiletin, metastasis, MMP, CREB, SP-1

## Abstract

Nobiletin, a polymethoxyflavone, has a few pharmacological activities, including anti-inflammation and anti-cancer effects. However, its effect on human osteosarcoma progression remains uninvestigated. Therefore, we examined the effectiveness of nobiletin against cellular metastasis of human osteosarcoma and the underlying mechanisms. Nobiletin, up to 100 μM without cytotoxicity, significantly decreased motility, migration and invasion as well as enzymatic activities, protein levels and mRNA expressions of matrix metalloproteinase (MMP)-2 and MMP-9 in U2OS and HOS cells. In addition to inhibition of extracellular signal-regulated kinase (ERK) and c-Jun N-terminal kinase (JNK), the inhibitory effect of nobiletin on the DNA-binding activity of the transcription factor nuclear factor-kappa B (NF-κB), cAMP response element-binding protein (CREB), and specificity protein 1 (SP-1) in U2OS and HOS cells. Co-treatment with ERK and JNK inhibitors and nobiletin further reduced U2OS cells migration and invasion. These results indicated that nobiletin inhibits human osteosarcoma U2OS and HOS cells motility, migration and invasion by down-regulating MMP-2 and MMP-9 expressions via ERK and JNK pathways and through the inactivation of downstream NF-κB, CREB, and SP-1. Nobiletin has the potential to serve as an anti-metastatic agent for treating osteosarcoma.

## INTRODUCTION

Osteosarcoma is the most prevalent and high-graded form of primary bone cancer, comprising approximately 60% of all bone sarcomas [1, 2]. In 1970s, surgery alone cured less than 20% of patients with osteosarcoma because the majority of deaths resulted from distant metastasis. Approximately 20% of patients with osteosarcoma will relapse within 5 years and have detectable metastasis at presentation and the lung is the predominant site of distant disease. Recently, the radical surgery and chemotherapy for osteosarcoma have advanced and improved survival rates to approximately 70% at 5 years, but the results remain unsatisfactory, particularly of those pertaining to cancer metastasis.

Metastasis is a complex process that includes several events collectively termed the invasion-metastasis cascade [3]. The coordination of several signaling pathways results in the detachment of tumor cells, motility, degradation of the extracellular matrix (ECM), invasion, migration, adhesion to endothelial cells, and the reestablishment of growth at a distant site [4]. Initially, the epithelial-mesenchymal transition (EMT) combines loss of epithelial cell junction proteins (e.g. E-cadherin) and the gain of mesenchymal markers, such as N-cadherin and Vimentin [5]. Degradation of ECM by proteolytic enzymes, such as matrix metalloproteinases (MMPs), plays a critical role in angiogenesis, tumor formation and progression, and microenvironment formation. ECM degradation by cancer cells through MMPs may disrupt the intercellular matrix to promote cancer cell mobility, eventually causing metastasis [6–8]. Among MMPs, MMP-2 (gelatinase A) and MMP-9 (gelatinase B) are vital enzymes involved in the degradation of gelatin, collagen and laminin, main components of ECM [9] and high expressions of them are indicators of high invasive potential of cancer cells and are related to poor prognosis of patients [3, 10, 11]. In contrast, down-regulation of MMP-2 and MMP-9 provides a preventive measure against tumor metastasis [12, 13]. Additionally, the 5′ end of promoter region of the MMP-2 and MMP-9 gene contains a variety of regulatory sequences that binds well-characterized transcriptional units of MMP-2 and MMP-9, including nuclear factor-kappa B (NF-κB), SP-1, cAMP response element-binding protein (CREB), and activator protein (AP)-1, possibly contributing to tumor metastasis [14, 15].

Recently, there is an increasing focus on naturally occurring plant products, that provides a preventive strategy for people with a high risk of cancers [16]. Flavonoids, a vast group of polyphenolic compounds ubiquitously present in plants [17], exhibit a wild spectrum of pharmacological properties and offer significantly protection against various cancers [18, 19]. As a member of flavonoids, nobiletin (5, 6, 7, 8, 3′ 4′-hexamethoxyflavone) has been proposed to have anti-cancer properties, including anti-angiogenesis, anti-proliferation, anti-metastasis and induced apoptosis *in vitro* and *in vivo* [20–23]. Accumulate studies have showed that nobiletin also suppressed the expression of MMPs, which may serve as a promising agent for suppression of cancer invasion and metastasis [24]. Moreover, nobiletin considerably attenuates metastasis via both ERK and PI3K/Akt pathway in HGF-treated liver cancer HepG2 cells, indicating the greater bioavailability of nobiletin than apigenin, tricetin and tangeretin. Therefore, nobiletin shows the higher potential of being developed for clinical applications [25]. However, the effects of nobiletin on human osteosarcoma migration and invasion, and the underlying mechanisms are not yet defined. In the present study, we investigated the potential inhibitory effects of nobiletin on osteosarcoma metastasis *in vitro* and also revealed the possible molecular mechanisms.

## RESULTS

### Cytotoxicity of nobiletin on U2OS, HOS and MC3T3-E1 cells

**Figure 1:**
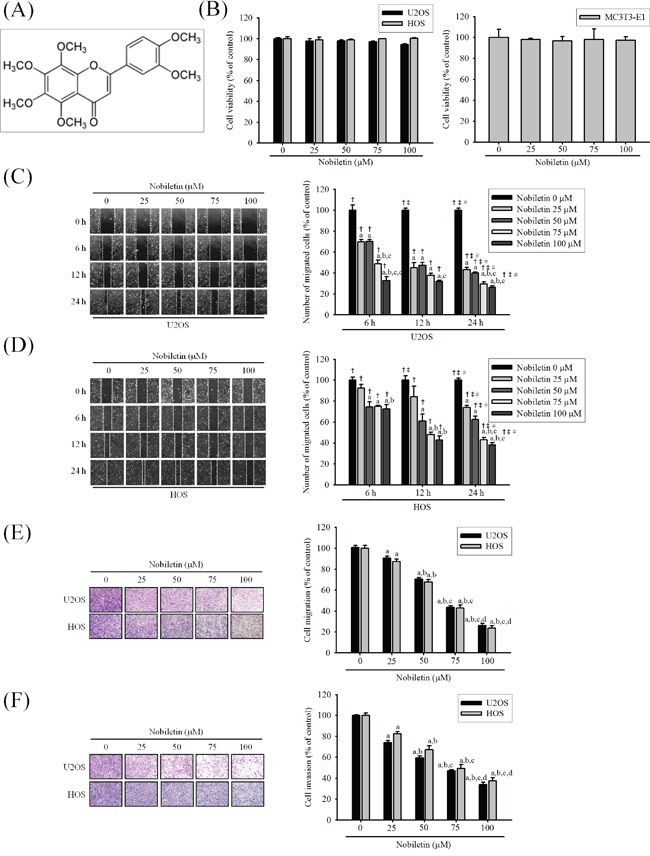
Nobiletin inhibits *in vitro* wound closure, migration and invasion in the U2OS and HOS cells **A.** The structure of nobiletin. **B.** U2OS and HOS human osteosarcoma cells and MC3T3-E1 mouse osteoblast cells were treated with nobiletin (0-100 μM) in serum free medium for 24 h by MTT assay are illustrated. **C.** and **D.** U2OS and HOS cells were wounded and then treated with nobiletin (0–100 μM) for 24 h in a serum-containing medium. At 0 h, 6 h, 12 h and 24 h, phase-contrast pictures of the wounds at four different locations were taken. **E.** Cell migration was measured using a Boyden chamber for 24 h with polycarbonate filters. **F.** Cell invasion was measured using a Matrigel-coated Boyden chamber for 48 h with polycarbonate filters. Cytotoxicity effects: U2OS: *F* = 2.827, *p* = 0.083; HOS: *F* = 0.274, *p* = 0.888; MC3T3-E1: *F* = 0.121, *p* = 0.972. Concentration effects: wounding healing (U2OS: *F* = 279.878, *p* < 0.001; HOS: *F* = 109.236, *p* < 0.001); cell migration (U2OS: *F* = 296.344, *p* < 0.001; HOS: *F* = 464.672, *p* < 0.001); invasion (U2OS: *F* = 327.312, *p* < 0.001; HOS: *F* = 196.642, *p* < 0.001). ^a^Significantly different, *p* < 0.05, when compared with 0 μM. ^b^Significantly different, *p* < 0.05, when compared with 25 μM. ^c^Significantly different, *p* < 0.05, when compared with 50 μM. ^d^Significantly different, *p* < 0.05, when compared with 75 μM. Time effects: wound healing (U2OS: *F* = 1555.239, p < 0.001; HOS: *F* = 928.975, *p* < 0.001). †Significantly different, *p* < 0.05, when compared with the vehicle group. ‡Significantly different, *p* < 0.05, when compared with 6 h. #Significantly different, *p* < 0.05, when compared with 12 h.

To determine the cytotoxic effects of nobiletin on human osteosarcoma cells and mouse osteoblast cells, the effects of serially diluted nobiletin on U2OS, HOS and MC3T3-E1 cells were examined using the MTT assay. Nobiletin (0-100 μM) did not effectively inhibit the cell viability of U2OS (*p* = 0.083), HOS (*p* = 0.888) and MC3T3-E1 (*p* = 0.972) cells after treatment for 24 h (Figure 1B). Therefore, we used this concentration range for nobiletin in the subsequent experiments involving osteosarcoma cells.

### Nobiletin inhibits cell invasion and migration of U2OS and HOS cells

As shown in Figure 1C and 1D, nobiletin dose- and time-dependently reduced U2OS and HOS cells moving into the wound (U2OS: *p* < 0.001; HOS: *p* < 0.001). Similarly, using a modified chamber with or without Matrigel, nobiletin markedly reduced migration (U2OS: *p* < 0.001; HOS: *p* < 0.001) and invasion (U2OS: *p* < 0.001; HOS: *p* < 0.001) activities in U2OS and HOS cells (Figure 1E and 1F). The results suggested that nobiletin significantly inhibits the motility, migration potential and invaseness of U2OS and HOS cells.

### Nobiletin reduces expressions and proteolytic activities of MMP-2 and MMP-9 of U2OS and HOS cells

**Figure 2:**
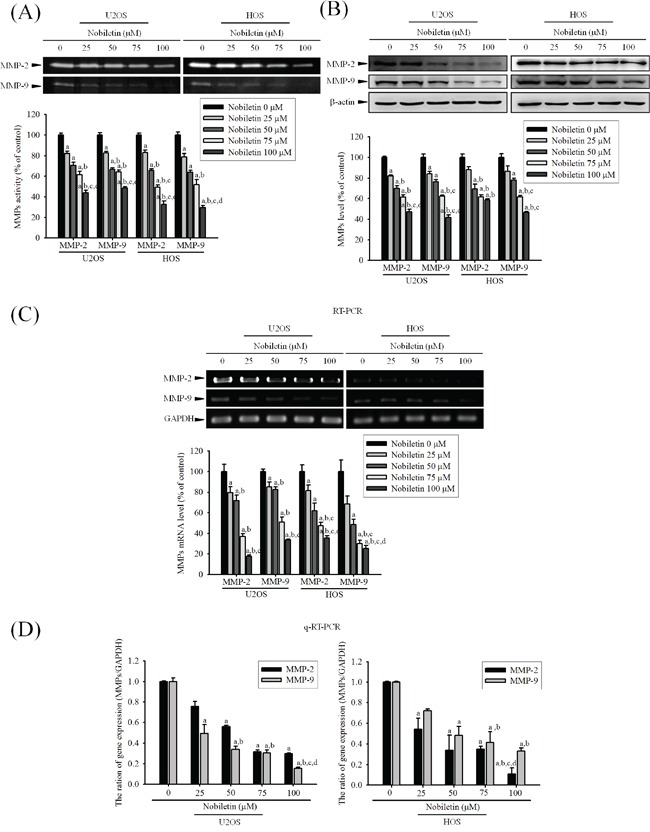
Nobiletin inhibits MMP-2 and MMP-9 proteolytic activity, protein and mRNA expression **A.** U2OS and HOS cells were treated with nobiletin (0–100 μM) for 24 h in a serum free medium and then subjected to gelatin zymography to analyze the activity of MMP-2 and MMP-9. **B.** Western blotting to analyze the protein levels of MMP-2 and MMP-9. Quantitative results of MMP-2 and MMP-9 protein levels, which were adjusted with β-actin protein level. **C.** RT-PCR and **D.** q-RT-PCR to analyze the mRNA expression of MMP-2 and MMP-9. Quantitative MMP-2 and MMP-9 mRNA levels were adjusted to GAPDH level. Concentration effects: gelatin zymography (MMP-2 of U2OS: *F* = 60.254, *p* < 0.001 and HOS: *F* = 160.282, *p* < 0.001; MMP-9 of U2OS: *F* = 133.253, *p* < 0.001 and HOS: *F* = 67.263, *p* < 0.001); western blot (MMP-2 of U2OS: *F* = 92.251, *p* < 0.001 and HOS: *F* = 25.151, *p* < 0.001; MMP-9 of U2OS: *F* = 25.156, *p* < 0.001 and HOS: *F* = 172.876, *p* < 0.001); RT-PCR (MMP-2 of U2OS: *F* = 68.584, *p* < 0.001 and HOS: *F* = 138.258, *p* < 0.001; MMP-9 of U2OS: *F* = 60.675, *p* < 0.001 and HOS: *F* = 63.82, *p* < 0.001); q-RT-PCR (MMP-2 of U2OS: *F* = 8.363, *p* = 0.019 and HOS: *F* = 116.834, *p* < 0.001; MMP-9 of U2OS: *F* = 186.999, *p* < 0.001 and HOS: *F* = 52.371, *p* < 0.001). ^a^Significantly different, *p* < 0.05, when compared with 0 μM. ^b^Significantly different, *p* < 0.05, when compared with 25 μM. ^c^Significantly different, *p* < 0.05, when compared with 50 μM. ^d^Significantly different, *p* < 0.05, when compared with 75 μM.

For clarifying whether MMP-2 and MMP-9 involves the nobiletin-induced suppression of cell motility and invasion, gelatin zymography was used. It showed that nobiletin dose-dependently reduced enzyme activities of MMP-2 (U2OS: *p* < 0.001; HOS: *p* < 0.001) and MMP-9 (U2OS: *p* < 0.001; HOS: *p* < 0.001) in U2OS and HOS cells (Figure 2A). Also, western blotting revealed that nobiletin progressively reduced protein expressions of MMP-2 (U2OS: *p* < 0.001; HOS: *p* < 0.001) and MMP-9 (U2OS: *p* < 0.001; HOS: *p* < 0.001) (Figure 2B). To further clarify the down-regulatory effects of nobiletin on protease, reverse transcription polymerase chain reaction (PCR) and quantitative PCR were conducted. Actually, nobiletin significantly reduced mRNA expressions of MMP-2 (RT-PCR—U2OS: *p* < 0.001; HOS: *p* < 0.001. q-RT-PCR—U2OS: *p* = 0.019; HOS: *p* < 0.001) and MMP-9 (RT-PCR—U2OS: *p* < 0.001; HOS: *p* < 0.001. q-RT-PCR—U2OS: *p* < 0.001; HOS: *p* < 0.001) dose-dependently in U2OS and HOS cells (Figure 2C and 2D). The results indicated that the nobiletin-induced antimetastatic effect is related to the inhibition of the degradative processes of tumor metastasis enzymatically in U2OS and HOS cells.

### Nobiletin reverses EMT by increasing E-cadherin and decreasing Vimentin expressions in U2OS and HOS cells

To corroborate the anti-metastatic effect of nobiletin on human osteosarcoma cells, we examined the EMT-related protein expression through western blotting. High (100 μM) dose of nobiletin effectively inhibited the Vimentin (U2OS: *p* = 0.004; HOS: *p* < 0.001) expression, but dose-dependently increased the E-cadherin (U2OS: *p* < 0.001; HOS: *p* < 0.001) expression (Supplementary Figure S1). Accordingly, the nobiletin-induced expression is associated with EMT reversal.

### Nobiletin suppresses the activity of NF-κB, CREB, and SP-1 of U2OS and HOS cells

**Figure 3:**
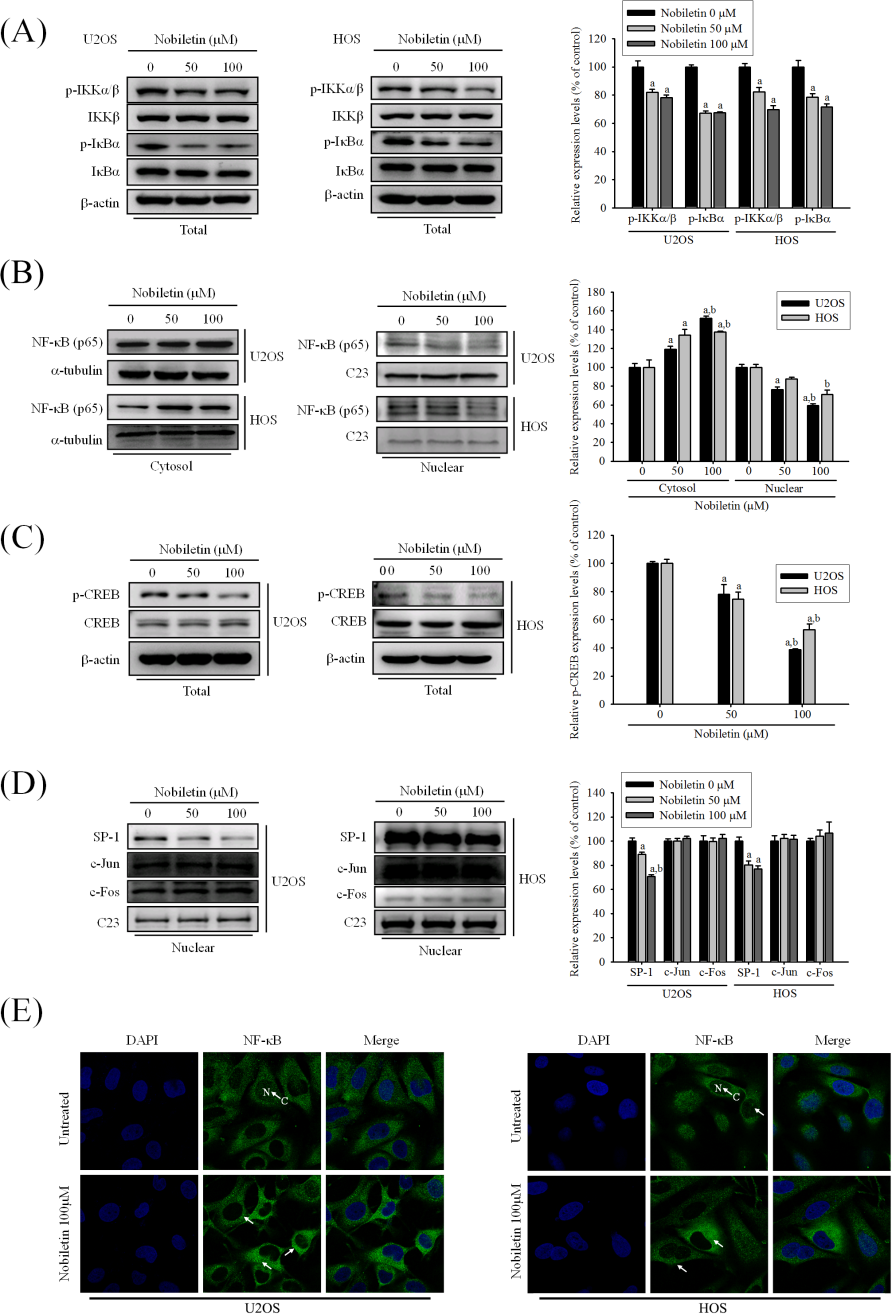
NF-κB and CREB are crucial to nobiletin-mediated transcriptional inhibition of MMP-2 and MMP-9 **A-D.** U2OS and HOS cells were treated with nobiletin (0–100 μM) for 24 h. The nuclear fraction and total cell lysates were prepared as described in Materials and Methods. Levels of NF-κB, SP-1, c-Jun and c-Fos from nuclear (B and D) and levels of p-IKKα/β, IKKβ, p-IκBα, IκBα, CREB and p-CREB from the total cell lysates (A and C) were determined by western blotting. Quantitative results of p-IKKα/β, p-IκBα, NF-κB, SP-1, c-Jun, c-Fos and p-CREB protein levels were adjusted with β-actin, C23 or α-tubulin protein level. Concentration effects: p-IKKα/β (U2OS: *F* = 15.419, *p* = 0.004; HOS: *F* = 28.397, *p* = 0.001); p-IκBα (U2OS: *F* = 194.788, *p* < 0.001; HOS: *F* = 21.407, *p* = 0.002); total-p-CREB (U2OS: *F* = 61.097, *p* < 0.001; HOS: *F* = 34.873, *p* < 0.001); cytosol-NF-κB (U2OS: *F* = 60.412, *p* < 0.001; HOS: *F* = 16.628, *p* = 0.007); nuclear-NF-κB (U2OS: *F* = 50.038, *p* < 0.001; HOS: *F* = 17.562, *p* = 0.003); SP-1 (U2OS: *F* = 58.954, *p* < 0.001; HOS: *F* = 49.681, *p* < 0.001); c-Jun (U2OS: *F* = 0.402, *p* =0.685; HOS: *F* = 0.097, *p* =0.909); c-Fos (U2OS: *F* = 0.124, *p* =0.885; HOS: *F* = 0.207, *p* =0.747). ^a^Significantly different, *p* < 0.05, when compared with 0 μM. ^b^Significantly different, *p* < 0.05, when compared with 25 μM. **E.** U2OS and HOS cells were stained for NF-κB by immunofluorescence. Pre-treatment with 100 μM nobiletin for 24 h significantly inhibited the nuclear translocation of NF-κB (White arrows were added to direct against distinct changes).

To further define the role of the nuclear translocation of NF-κB and other transcription factors SP-1, CREB and AP-1, we examined the NF-κB expression in the cell cytosolic and nuclear fractions, and IKK dependent pathway through western blotting. In U2OS and HOS cells, nobiletin considerably reduced the phosphorylation of p-IKKα/β (U2OS: *p* = 0.004; HOS: *p* = 0.001) and p-IκBα (U2OS: *p* < 0.001; HOS: *p* = 0.002) (Figure 3A), and protein expression of NF-κB in the cell nuclear (U2OS: *p* < 0.001; HOS: *p* = 0.003) fraction with the concomitant increase of the NF-κB expression in the cytosolic (U2OS: *p* < 0.001; HOS: *p* = 0.007) fraction (Figure 3B). Nobiletin down-regulated the p-CREB (U2OS: *p* < 0.001; HOS: *p* < 0.001) and the SP-1 (U2OS: *p* < 0.001; HOS: *p* < 0.001) expressions in the nuclear fraction in U2OS and HOS cells, whereas nobiletin did not affect c-Jun (U2OS: *p* = 0.685; HOS: *p* = 0.909) and c-Fos (U2OS: *p* = 0.885; HOS: *p* = 0.747) expressions (Figure 3C and 3D). Furthermore, the inhibitory effect on nuclear translocation of NF-κB from the cytoplasm was observed in U2OS and HOS cells using immunocytofluorescence (Figure 3E). Thus, the transcriptional inhibition of MMP-2 and MMP-9 may down-regulate NF-κB and CREB, but not AP-1.

### Nobiletin reduces promoter activities of MMP-2 and MMP-9 of U2OS and HOS cells

**Figure 4:**
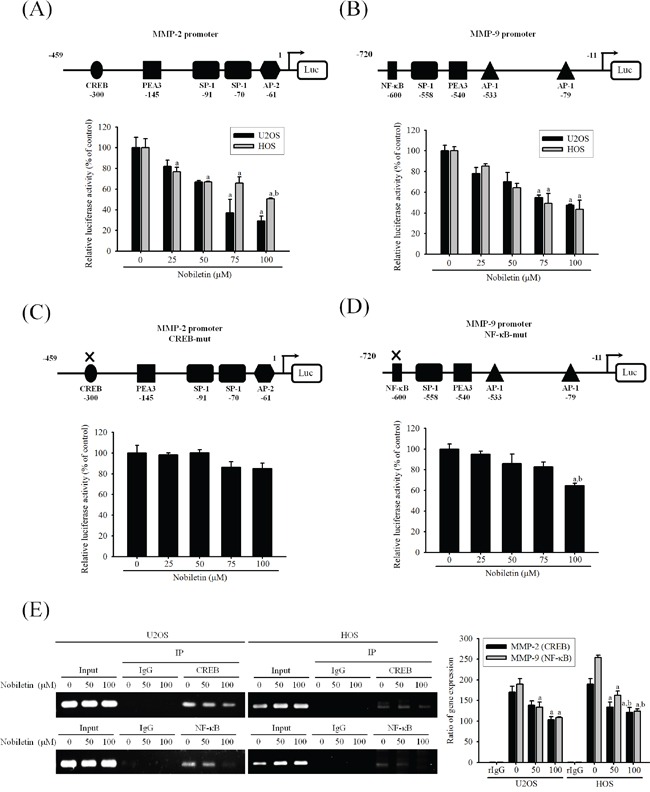
Nobiletin inhibits MMP-2 and MMP-9 promoter and DNA-binding activity **A-D.** U2OS and HOS cells were treated with nobiletin (0–100 μM) for 24 h and then subjected to luciferase assay to analyze the promoter activity of MMP-2 and MMP-9. Upper: schematic of the wild type promoter region of the human MMP-2 and MMP-9 gene and the utilized mutant constructs. Lower: (A and B) U2OS and HOS cells were transfected with MMP-2 and MMP-9 promoter plasmid (wild type); (C and D) U2OS cells were transfected with MMP-2 (CREB-mut) and MMP-9 (NF-κB-mut) promoter plasmid. Transfected cells were treated with nobiletin (0–100 μM) for 24 h. Luciferaes activity was determined in triplicates, was normalized to the β-galactosidase. Concentration effects: promoter activity (wt-MMP-2 of U2OS: *F* = 13.634, *p* = 0.007 and HOS: *F* = 25.179, *p* = 0.002; wt-MMP-9 of U2OS: *F* = 14.477, *p* = 0.006 and HOS: *F* = 13.898, *p* = 0.006); CREB-mut MMP-2: *F* = 4.612, *p* = 0.062; NF-κB-mut MMP-9: *F* = 12.477, *p* = 0.008. ^a^Significantly different, *p* < 0.05, when compared with 0 μM. ^b^Significantly different, *p* < 0.05, when compared with 25 μM. **E.** ChIP assay was used to analyze the association of transcription factor CREB with MMP-2 and NF-κB with MMP-9 promoter region in U2OS and HOS cells. Concentration effects: CREB (MMP-2 of U2OS: *F* = 9.09, *p* = 0.015 and HOS: *F* = 72.564, *p* < 0.001); NF-κB (MMP-9 of U2OS: *F* = 14.909, *p* = 0.005 and HOS: *F* = 77.244, *p* < 0.001). ^a^Significantly different, *p* < 0.05, when compared with 0 μM. ^b^Significantly different, *p* < 0.05, when compared with 50 μM.

We further identify whether nobiletin suppresses the transcription factors binding to the promoter of MMP-2 and MMP-9 to regulate their gene expressions in U2OS and HOS cells. The luciferase reporter assay revealed that nobiletin dose-dependently suppressed the promoter activities of MMP-2 (U2OS: *p* = 0.007; HOS: *p* = 0.002) and MMP-9 (U2OS: *p* = 0.006; HOS: *p* = 0.006) in U2OS and HOS cells, indicating the nobiletin-induced inhibition of MMP-2 and MMP-9 expressions at the transcriptional level (Figure 4A and 4B). Next, we assessed the single-site mutation plasmids of CREB and NF-κB and their promoter activities. In U2OS cells, nobiletin at high concentrations (75 and 100μM) seemed to inhibit MMP-2-CREB-mut but without significance (*p* = 0.062). Intriguingly, we observed that a high concentration (100 μM) of nobiletin inhibited MMP-9-NF-κB-mut (*p* = 0.008) (Figure 4C and 4D). For exploring whether nobiletin inhibites the binding activity of NF-κB and CREB, the chromatin immunoprecipitation (ChIP) assay was performed. We found that 50 and 100 μM nobiletin reduced the activity of CREB (U2OS: *p* = 0.015, HOS: *p* < 0.001) and NF-κB (U2OS: *p* < 0.005, HOS: *p* < 0.001) at the binding target sequence of MMP-2 and MMP-9 promoters, especially in HOS cells dose-dependently (Figure 4E). These results provided strong evidence that nobiletin prevents the binding activity of the target sequence of CREB and NF-κB to down-regulate transcription levels of MMP-2 and MMP-9 in U2OS and HOS cells.

### Nobiletin suppresses cell migration and invasion by down-regulating phosphorylation of ERK and JNK of U2OS and HOS cells

**Figure 5:**
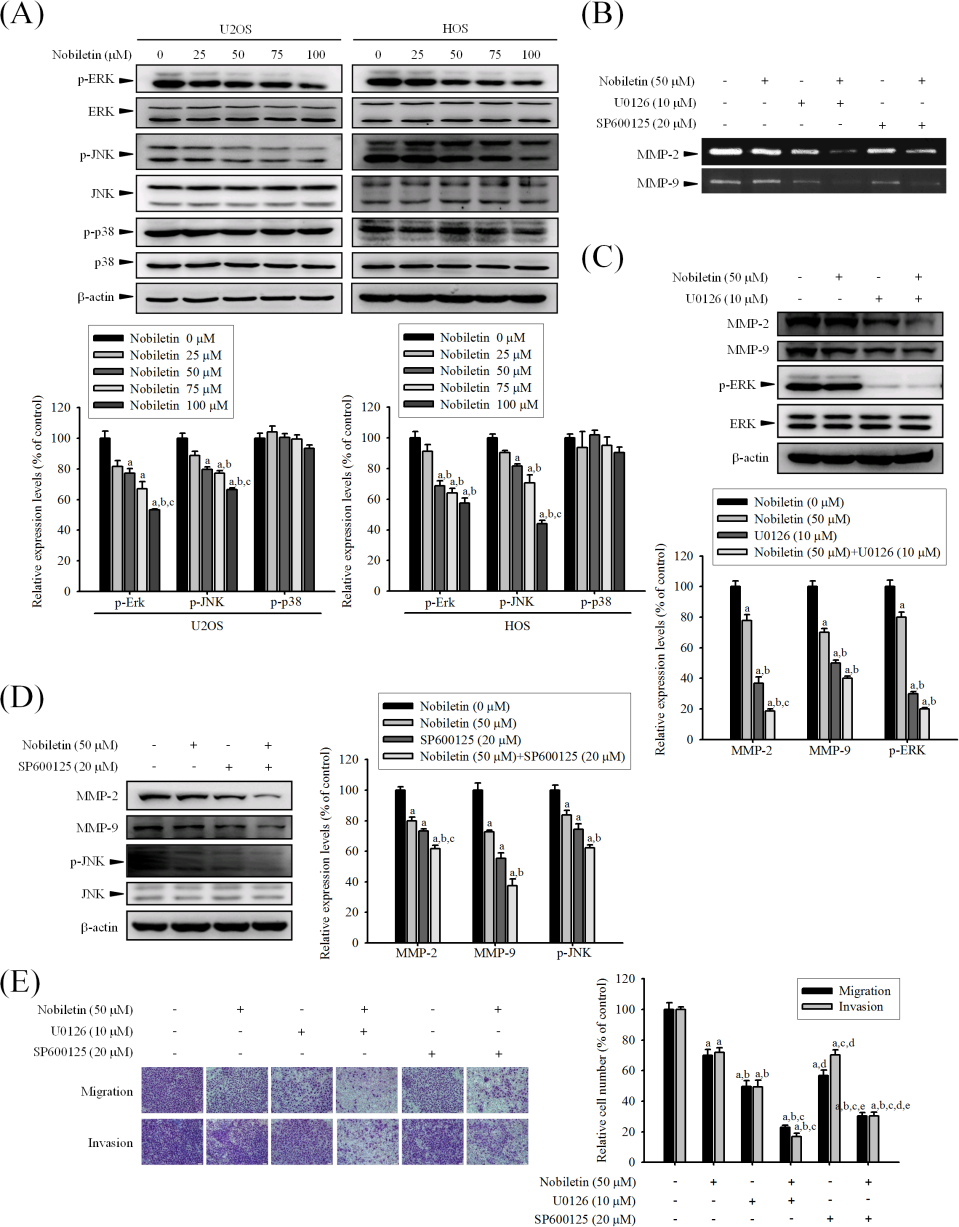
Effects of nobiletin on the MAPK pathway in U2OS and HOS cells **A.** U2OS and HOS cells were treated with nobiletin (0–100 μM) for 24 h and the total cell lysates were then subjected to western blotting with anti-ERK, anti-JNK and anti-p38 antibodies. Quantitative results of ERK, JNK and p38 protein levels were adjusted to β-actin protein level. Concentration effects: p-ERK (U2OS: *F* = 22.386, *p* < 0.001; HOS: *F* = 25.074, *p* < 0.001); p-JNK (U2OS: *F*= 31.325, *p* < 0.001; HOS: *F*= 53.906, *p* < 0.001); p-p38 (U2OS: *F* = 1.694, *p* =0.227; HOS: *F* = 0.687, *p* =0.617). ^a^Significantly different, *p* < 0.05, when compared with control. ^b^Significantly different, *p* < 0.05, when compared with 25 μM. ^c^Significantly different, *p* < 0.05, when compared with 50 μM. **B-E.** U2OS cells were co-treated with specific protein inhibitor U0126 or SP600125, which incubated in the presence or absence of nobiletin (50 μM) for 24 h. (B) Gelatin zymography to analyze the activity of MMP-2 and MMP-9. (C and D) U2OS cells were co-treated with specific protein inhibitor U0126 or SP600125, which incubated in the presence or absence of nobiletin (50 μM) for 24 h and then the total cell lysates were subjected to western blotting with (C) anti-MMP-2, anti-MMP-9 and anti-ERK. MMP-2: *F* = 122.929, *p* < 0.001; MMP-9: *F* = 104.197, *p* < 0.001; p-ERK: *F* = 186.714, *p* < 0.001. (D) anti-MMP-2, anti-MMP-9 and anti-JNK. MMP-2: *F* = 56.897, *p* < 0.001; MMP-9: *F* = 50.368, *p* < 0.001; p-ERK: *F* = 26.765, *p* < 0.001. ^a^Significantly different, *p* < 0.05, when compared with 0 μM. ^b^Significantly different, *p* < 0.05, when compared with noibletin-treated group. ^c^Significantly different, *p* < 0.05, when compared with U0126 or SP600125 treated-group. (E) Cell migration and invasion ability of U2OS cells were measured by using a Boyden chamber assay. Migration: *F* = 68.12, *p* < 0.001; invasion: *F* = 102.991, *p* < 0.001. ^a^Significantly different, *p* < 0.05, when compared with 0 μM. ^b^Significantly different, *p* < 0.05, when compared with noibletin-treated group. ^c^Significantly different, *p* < 0.05, when compared with U0126 treated-group. ^d^Significantly different, *p* < 0.05, when compared with nobiletin plus U0126-treated group. ^e^Significantly different, *p* < 0.05, when compared with SP600125 treated-group.

To assess whether nobiletin mediates phosphorylation of the three major mammalian MAPK pathways, the effects of nobiletin on the constitutive activation status of ERK, JNK, and p38 were analyzed through western blotting. Nobiletin markedly reduced p-JNK (U2OS: *p* < 0.001; HOS: *p* < 0.001) and p-ERK (U2OS: *p* < 0.001; HOS: *p* < 0.001); however, it did not affect the p-p38 (U2OS: *p* = 0.227; HOS: *p* = 0.617) activity in U2OS and HOS cells, and the protein levels of total JNK, ERK, and p38 remained unchanged (Figure 5A). To further investigate whether nobiletin mediates the inhibition of cell invasion and migration as well as MMP-2 and MMP-9 secretions mainly by inhibiting JNK and ERK phosphorylation, specific inhibitors of ERK (U0126) and JNK (SP600125) were used in U2OS cells. Zymography revealed that co-treatment with inhibitors and nobiletin further reduced the proteolytic activities of MMP-2 and MMP-9 (Figure 5B). A similar effect was observed through western blotting (MMP-2: *p* < 0.001; MMP-9: *p* < 0.001; p-ERK: *p* < 0.001; p-JNK: *p* < 0.001) in U2OS cells (Figure 5C and 5D). As shown in Figures 5E and Supplementary Figure S2, co-treatment with inhibitors and nobiletin further reduced wound closure (*p* < 0.001), invasion (*p* < 0.001) and migration (*p* < 0.001) of U2OS cells compared with individual treatments, indicating that the inhibition of MMP-2 and MMP-9 to suppress U2OS cells motility, invasion and migration was via down-regulation of the ERK and JNK pathways.

## DISCUSSION

**Figure 6:**
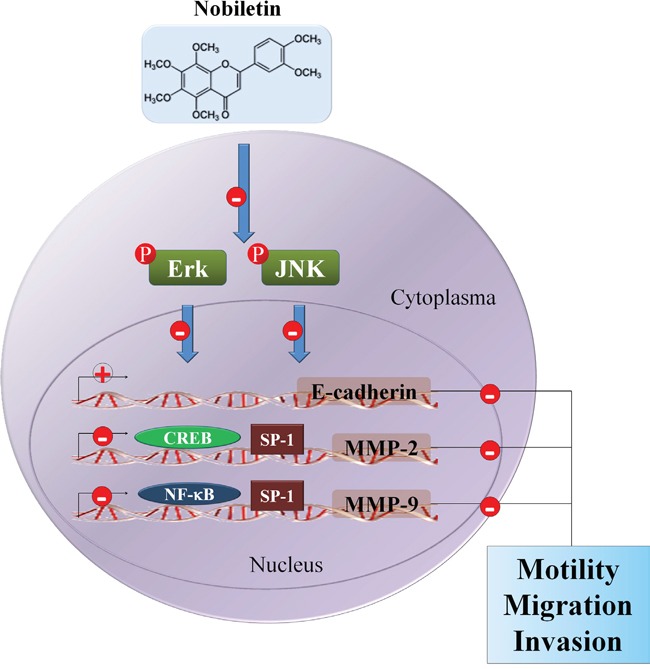
Propose signaling pathways by which nobiletin inhibits cell motility, migration and invasion of human osteosarcoma cells ⊝ indicates inhibition.

The present study sought to determine the anti-metastatic effect of nobiletin on the human osteosarcoma cells *in vitro* by monitoring the regulation of MMP-2 and MMP-9 expressions and the possible signaling pathways (Figure 6). The major findings were as follows: (a) nobiletin at concentrations (up to 100 μM) without cytotoxicity inhibited human osteosarcoma U2OS and HOS cells motility, migration potential and invasiveness, accompanied by a decrease of MMP-2 and MMP-9 expressions; (b) nobiletin reversed EMT by increasing E-cadherin and decreasing Vimentin expressions in U2OS and HOS cells; (c) nobiletin suppressed the activities of NF-κB, CREB, and SP-1 to reduce MMP-2 and MMP-9 promoter activities in U2OS and HOS cells and (d) nobiletin suppressed cell migration and invasion by down-regulating phosphorylation of ERK and JNK in U2OS and HOS cells.

In human acute myeloid leukemia, gastric cancer and hepatocellular carcinoma, nobiletin significantly suppresses cell proliferation and induces apoptosis by inducing caspase signals and down-regulating the expressions of Bcl-2 and Cox-2 [18, 19, 23]. However, we found that nobiletin at low concentrations (up to 100 μM) without cytotoxicity dose-dependently reduced the cell motility, invasion and migration of U2OS and HOS cells. Similarly, such effect has been reported that nobiletin markedly reduces the migration ability of breast cancer cells under noncytotoxic concentrations [26]. In general, nobiletin is considered as a flavonoid with weak cytostatic properties (up to 100 μM) towards progression of cancer cells *in vivo* and *in vitro* [22, 24, 27]. In the present study, we used the concentration range 0-100 μM of nobiletin in all subsequent experiments. Moreover, pharmacokinetic study in rats for orally administered nobiletin (2 mg/kg) has been reported that after oral administration of nobiletin, the Cmax of plasma concentration was calculated to be 4.7 ± 0.5 ng/mL [28]. Therefore, more animal studies and clinical trials using the concentration range of nobiletin are needed to further justify its clinical value.

During invasion, MMPs facilitate the degradation and invasion of ECM components, and participate in the onset and progression of tumors [11, 29]. It is well-established that inhibitions of MMP gene expression or enzyme activity are early targets for preventing cancer metastasis [30]. Of all MMPs, MMP-2 and MMP-9 are most widely studied that both gain and loss-of function are associated with osteosarcoma progression [31–34]. As reported by others about other flavonoids such as naringin and isorhamnetin, nobiletin could inhibit MMP-2 and MMP-9 expressions as well as protease activities in U2OS and HOS cells to suppress migration and invasion abilities [35, 36]. Because E-cadherin is regarded as a gatekeeper of the epithelial state in various epithelial cell types and the inhibition of E-cadherin expression involves osteosarcoma invasion and metastasis, EMT is part of the process of cancer cell dissemination and transmigration [37]. Interestingly, we observed that nobiletin reversed the EMT by suppressing Vimentin at a high concentration (100 μM) and dose-dependently increasing E-cadherin expressions. Previous study also reveals that nobiletin effectively attenuated hypoxia-induced epithelial-mesenchymal transition of human H1299 lung cancer cells [38]. Besides, loss of cell–cell connections, epithelial characteristics and remodeling of extracellular matrix mediated by MMPs, has been acknowledged as a critical mechanism for cancer metastasis. Thus, EMT reversal is considered as an efficient process of inhibiting tumor metastasis [39]. Moreover, some researchers have shown that EMT-related proteins have a close relationship with MMP-2 and MMP-9 [40, 41], so we inferred that nobiletin might inhibit the metastasis ability of U2OS and HOS cells by decreasing MMP-2 and MMP-9 protein expressions and then the EMT-related proteins might be also regulated by MMP-2 and MMP-9. Therefore, the down-regulation of MMP-2 and MMP-9 is related to the inhibition of osteosarcoma cell metastasis and is partly attributed to EMT reversal.

Since the activation of transcription factors to the promoter is primarily involved in the induction and maintenance of EMT and the diverse aspect of cancer biology, identification of these regulatory elements might be manipulated to reduce MMPs expression for affecting cancer metastasis [42–44]. Generally, a crucial c-Jun and c-Fos binding site, AP-1 is located in the promoter region of inducible type of MMP genes. Blocking in the AP-1 binding activity prevents tumor promoter-induced MMP-9 transcription [45]. Besides, NF-κB and SP-1 transcription factors also play a co-activators role in the regulation of MMP-2 and MMP-9 [46, 47]. However, once the IKK complex phosphorylates serine residues of IκB, resulting in NF-κB release and then translocates to the nucleus [48]. Researchers have reported that IKK/NF-κB pathway implicated in cancer metastasis [49, 50]. Thus, we postulated that nobiletin mediated reduction of MMP-2 and MMP-9 expressions to inhibit U2OS and HOS cellular migration and invasion may be deeply related to the suppression of DNA-binding activity of these regulatory factors. Consequently, nobiletin inhibited phosphorylation of IKKα/β and IκBα and the nuclear translocation of NF-κB. Moreover, nobiletin effectively suppressed SP-1 and CREB expressions, these findings are consistent with those in reports on leukemia cells [51], cholangiocarcinoma cells [52] and osteosarcoma cells [53], implicating the importance of NF-κB and CREB in the regulation of MMP-2 and MMP-9 in U2OS and HOS. Although c-Jun and c-Fos have been thought to be indispensable for the inhibition of MMP gene expression and both require synergistic cooperation with either the NF-κB or the SP-1 site, nobiletin had no effect on c-Jun and c-Fos in U2OS and HOS cells. It has been documented that NF-κB is critically important for MMP-9 to mediate cancer cell invasion, and the binding of CREB to promoter is centrally involved in MMP-2 gene expression and related to the malignancy of cancer cells [54]. We, therefore, used mutated NF-κB and CREB reporter plasmid and further demonstrated that this inhibitory effect of nobiletin on MMP-2 and MMP-9 activity is partially reversed in U2OS cells. Although the inhibitory effect on MMP-9 promoter activity was found in high concentration (100 μM) of nobiletin, we still supposed that the tendency to a decreased expression of SP-1 may explain the slight decrease in the MMP-9 level.

The MAPK pathways are prominent mechanisms that coordinate the transcription factors, such as NF-κB and CREB, which are activated and phosphorylated by ERK and JNK kinase systems to regulate downstream MMP-2 and MMP-9 gene expression, subsequently increase tumor initiation and progression [55–57]. Review studies documented the importance of ERK and JNK protein, which phosphorylate multiple transcriptional elements to promote cell proliferation, survival and invasion [58, 59]. Constitutive ERK activation increasing cell survival and cell invasion is influenced by integrin, which binds and activates the MAPK to down-regulate the E-cadherin adhesion molecule [60, 61]. However, our data demonstrated that nobiletin inhibited the phosphorylation of ERK and JNK, leading to the down-regulation of MMP-2 and MMP-9 expression and reversed EMT by increasing E-cadherin expression to decrease U2OS and HOS cellular migration and invasion. Previous studies shown that nobiletin inhibits invasion and migration capacities of nasopharyngeal carcinoma cell lines and glioma cell lines through down-regulating ERK and/ or JNK pathway [62, 63]. Nobiletin also suppresses MMP-9 expression by reducing p38 MAPK activity [64]. Furthermore, we were interested in nobiletin's inhibitory mechanism on ERK and JNK signaling pathway that mediates MMP-2 and MMP-9 expression, implicating in U2OS and HOS cells metastasis. Co-treatment with nobiletin and U0126 or SP600125 could further suppress MMP-2 and MMP-9 expressions and cell motility, invasion and migration in U2OS cells, suggesting that nobiletin inhibits U2OS and HOS cells metastasis through ERK and JNK pathways.

To summarize, this is the first study verifying the effects of nobiletin on human osteosarcoma cell metastasis. Nobiletin inhibits osteosarcoma cell metastasis by suppressing the expression and binding activities of NF-κB and CREB on MMP-2 and MMP-9 promoters. Furthermore, this inhibitory effect may down-regulate phosphorylation of ERK and JNK, reduce MMP-2 and MMP-9 expressions and partially reverse the EMT. Thus, nobiletin is a promising candidate for preventing osteosarcoma progression; however, clarifying the cellular and molecular mechanisms underlying tumor metastasis in the future may provide new therapeutic strategies.

## MATERIALS AND METHODS

### Cell culture and treatment

Human osteogenic sarcoma U2OS (female; 15 years old) and HOS (female; 13 years old) cells both obtained from the Food Industry Research and Development Institute (Hsinchu, Taiwan) were cultured in Dulbecco's Modified Eagle Medium and Eagle's MEM (Gibco BRL, Grand Island, NY, USA); mouse osteoblast MC3T3-E1 cell was obtained from ATCC and cultured in Alpha Modification of Eagle's Medium (Gibco BRL, Grand Island, NY, USA) supplemented with 10% fetal bovine serum (HyClone Laboratories, Inc. South Logan, Utah, USA), penicillin (100 U/mL) and streptomycin (100 μg/mL) in a humidified 37°C incubator with atmosphere of 5% CO2. Adhering cells were detached by incubation with trypsin. After reaching confluence, the cells were treated with appropriate amounts of stock solution (0.1 M in DMSO) of nobiletin (Enzo Life sciences. Ann Arbor, MI, USA) and then incubated for indicated time periods. The harvested culture medium and cells lysate were stored at −20°C until use.

### Analysis of cell viability [microculture tetrazolium (MTT) assay]

To evaluate the cytotoxicity of nobiletin, a MTT [3-(4, 5-dimethylthiazol-2-yl)-2, 5-diphenyl-tetrazolium bromide] assay was performed to determine the cell viability. Briefly, U2OS and HOS cells were seeded at a density of 8×10^4^ per well in a 24-well, and MC3T3-E1 cells were seeded at a density of 10^5^ per well in a 96-well plate for 16 h. Then, cells were treated with nobiletin at indicated concentrations (0, 25, 50, 75, 100 μM) for additional 24 h. Each concentration was repeated three times. After the exposure period, the medium was removed and followed by washing the cells with PBS. Then, the medium was changed and incubated with MTT solution (0.5 mg/ml) (Sigma, St. Louis, MO, USA) for 4 h. After removing the medium, the viable cell number per dish was directly proportional to the production of formazan which was solubilized in isopropanol and measured spectrophotometrically at 563 nm. The percentage of viable cells was estimated by comparing with the untreated control cells.

### Wound healing assay

We plated 8×10^5^ U2OS and HOS cells in 6 cm plates for 16 h and the cells were followed by starvation overnight. Wounded by scratching with yellow tip, the cells were incubated with condition medium containing 0.5% FBS and then received various treatments of nobiletin (0, 25, 50, 75, 100 μM) for 0, 6, 12, 24 h. Cells were co-treated with specificity protein inhibitor U0126 (ERK inhibitor) or SP600125 (JNK inhibitor) for 0, 24 h, and they were photographed using a phase-contrast microscope (Olympus CX41).

### Cell invasion and migration assay

The tumor invasion assay was performed using a modified Boyden chamber (Neuro Probe, Cabin John, MD) as described elsewhere [65]. Matrigel (Collaborative Biomedical Products, Bedford, MA) was diluted with cold filtered distilled water to receive the concentration 25 μg/50 μl and applied to the upper to 8 μm pore size polycarbonate membrane filters of the upper well. Briefly, U2OS and HOS cells were treated with indicated concentrations (0, 25, 50, 75, 100 μM) of nobiletin for 24 h. The migrated cells passed through the basement membrane layer and clung to the bottom of the Boyden chamber membrane, while non-invasive cells stayed in the upper chamber. The data were presented as the average number of cells attached to the bottom surface from randomly chosen files under a light microscope. In order to measure the ability of osteosarcoma cells on migration, cells were seeded into the upper well of Boyden chamber which were not coated with Matrigel. Migration of cells treated with different concentrations of nobiletin was measured as described in the cell invasion assay.

### Gelatin zymography

MMP-2 and MMP-9 activities were evaluated by gelatin zymography. Osteosarcoma cells treated with different concentrations (0, 25, 50, 75, 100 μM) of nobiletin for 24 h after plating 8×10^4^ cells in 24-well plates for 16 h. The harvested culture medium (16 μl) containing 10 μg of total protein was subjected to SDS-PAGE containing 0.1% gelatin. After electrophoresis, the gels was washed with washing buffer (2.5% Triton X100) to remove SDS and then incubated at 37°C in reaction buffer (40 mM Tris-HCl, 10 mM CaCl_2_, 0.01% NaN_3_). The proteolytic activity of MMP-2 and MMP-9 was performed by staining with Coomassie Brilliant Blue R-250. The intensity of unstained bands on blue background was measured by spot density measurement using a densitometer (AlphaImager 2000, Alpha Innotech Corp, San Leandro, CA).

### Western blot analysis

After the treatment with different concentrations (0, 25, 50, 75, 100 μM) of nobiletin, western blotting was conducted as described elsewhere [66]. The blot was subsequently operated with standard procedures and probed with the following primary antibodies: MMP-2, MMP-9, ERK, JNK, phospho-ERK, phospho-JNK, phospho-p38, IKKβ, phospho-IKKα/β, IκBα, phospho-IκBα and phospho-CREB (Cell Signaling Technologies, Danvers, MA); vimentin, NF-κB, C23, SP-1, CREB, c-Jun and c-Fos (Santa Cruz Biotechnology, California, USA); E-cadherin, p-38 and β-actin (BD biosciences, Bedford, MA, USA). Protein expression was detected by chemiluminescence with an ECL detection kit (Millipore, Bedford, MA, USA).

### Preparation of nuclear fraction

We plated 6×10^5^ U2OS and HOS cells in 6 cm plates for 16 h, followed by the treatment with different concentrations (0, 25, 50, 75, 100 μM) of nobiletin for 24 h. Nuclear extracts were obtained as Sigma-Aldrich procedure described. The cells were lysed with ice-cold buffer A (10 mM Hepes pH 7.9, 1.5 mM MgCl2, 10 mM KCL, 1 mM DTT, 0.5 mM PMSF, plus protease and phosphatase inhibitors), followed by dounce homogeniser and microcentrifuge to shear the cytoplasmic membranes. Cytoplasmin extracts were on the supernatant, resuspended pellet in cell lysis buffer (*iNtRON Biotechnology*, *INC*), and pipetted up and down to achieve homogenous mix. Followed by centrifugation at 13000 rpm for 30 min at 4°C in a microcentrifuge, we harvested the nuclear extract from the supernatant and stored at −20°C. The total and extracts protein were determined by BCA assay.

### RT-PCR and quantitative real-time PCR

Total RNA was isolated from U2OS and HOS cells using Total RNA Mini Kit (*Geneaid Biotech* Ltd.), according to the manufacturer's instructions. Total RNA (2 μg) was used to reverse transcribed into 20 μl cDNA by ABI-High-Capacity RNA-to-cDNA^TM^ Kit (Applied Biosystems inc. Foster City, California). The PCR was performed in mixture containing 2 μl RT buffer, 2 μl RT random primer, 0.8 μl dNTP mix (100 mM), 1 μl multi-scribe^TM^ reverse transcriptase. The appropriate primers (MMP-2, MMP-9 and GAPDH) were used for amplification of these genes, GAPDH was served as an internal control. The following forward (F) primers, and reverse (R) primers were used: MMP-2-F: ggCATCCAggTTATCggggA, R: ggCCCTgTCACTCCTgAgAT; MMP-9-F: CgggTgTAgAgTCTCTCgCT, R: CAACATCACCTATTggATCC; GAPDH-F: AgCCTTCTCCATggTTggTgAAgAC, R: CggAgTCAACggATTTggTCgTAT. Q-PCR was carried out using ABI StepOne Real-Time PCR System (Applied Biosystems, Foster City, CA, USA). The relative gene expressions of MMP-2 and MMP-9 were normalized to glyceraldehydes 3-phosphate dehydrogenase (GAPDH).

### Immunocytofluorescence

Cells were cultured on glass coverslips and fixed with 4% paraformaldehyde at room temperature for 20 min followed by PBS washing for three times. The cells were permeabilized with 0.5% Triton X-100 for 10 min. After washing with PBS, cells were blocked with 5% bovine serum albumin in PBS for 1 h. Then the samples were incubated with rabbit-anti-NF-κB antibody (1:200) at 4°C overnight. Subsequently, the samples were then washed by PBS and incubated with FITC-conjugated rabbit antibody for 1 h at room temperature. The nuclei were counterstained with DAPI for 5 min. The coverslips were then washed extensively and mounted on glass slides with mounting medium (DAKO, Glostrup, Denmark) for quantitative image analysis. The image of samples coverslips were examined with Zeiss LSM 510 META confocal microscope.

### Luciferase reporter assay

We plated 8×10^4^ U2OS and HOS cells in 24-well plate for 16 h. Followed by plasmid DNA transfection. pGL3-MMP-2 promoter (wild-type or CREB-mut) or MMP-9 promoter (wild-type or NF-κB-mut) plasmid was transfected into U2OS and HOS cells using lipofectamine 2000 (Invitrogen Life Technologies, Carlsbad, CA, USA) according to the manufacturer's instructions. After incubation, cultured cells were treated with different concentrations (0, 25, 50, 75, 100 μM) of nobiletin for 24 h. Cells were harvested and lysed by reporter lysis buffer. For quantification of luciferase and β-galactosidase activity, cell lysates were subjected to luciferase assay using a Luciferase Assay system. Respectively, luciferase activity was normalized to β-galatosidase activity to account for the transfection efficiency.

### Chromatin immunoprecipitation (ChIP) assay

The CHIP assay procedure was performed as described elsewhere [67]. Briefly, Protein-DNA complexes were cross-linked using formaldehyde at a final concentration of 1% at room temperature for 10 min. Cells were washed twice with ice-cold PBS and were collected by centrifugation at 4°C, and resuspended in the cell lysis buffer containing 50 mM Tris–HCl, pH 8, 10 mM EDTA, 1% SDS, and protease inhibitors. Cell lysates were sonicated to give a DNA size of approximately 300 bp, and supernatants were diluted with dilution buffer (16.7 mM Tris–HCl, pH 8, 1% Triton X-100, 1.2 mM EDTA, 167 mM NaCl, 0.01% SDS, and protease inhibitors). Immune complexes were precleared with salmon sperm DNA/protein G agarose slurry and then treated with antibody overnight at 4°C. DNA immunoprecipitated with antibodies specific to NF-κB, CREB or the control, rabbit immunoblobulin G (IgG), was purified and extracted using QIAquick PCR purification kit (Qiagen), according to the manufacturer's instructions. The specific primer of NF-κB and CREB of human MMP-2 and MMP-9 promoter binding region were used for PCR amplification.

### Statistical analysis

The values are presented as the mean ± SE from three independent experiments. For all data statistical analysis, the significant differences between the control group and the experiment group were performed using one-way ANOVA with post hoc Bonferroni test. Results were considered to be statistically significant only if the *p* value < 0.05 (Sigma-Stat 2.0, Jandel Scientific, San Rafael, CA, USA).

## SUPPLEMENTARY FIGURES


