# Supplementary material for: Nobiletin inhibits human osteosarcoma cells metastasis by blocking ERK and JNK-mediated MMPs expression
Source: Oncotarget. 2016 Apr 29;7(23):35208–23. doi: 10.18632/oncotarget.9106 (PMC5085222; doi:10.18632/oncotarget.9106)
Supplement: Supplementary file 1 [file oncotarget-07-35208-s001.pdf]

SUPPLEMENTARY FIGURES

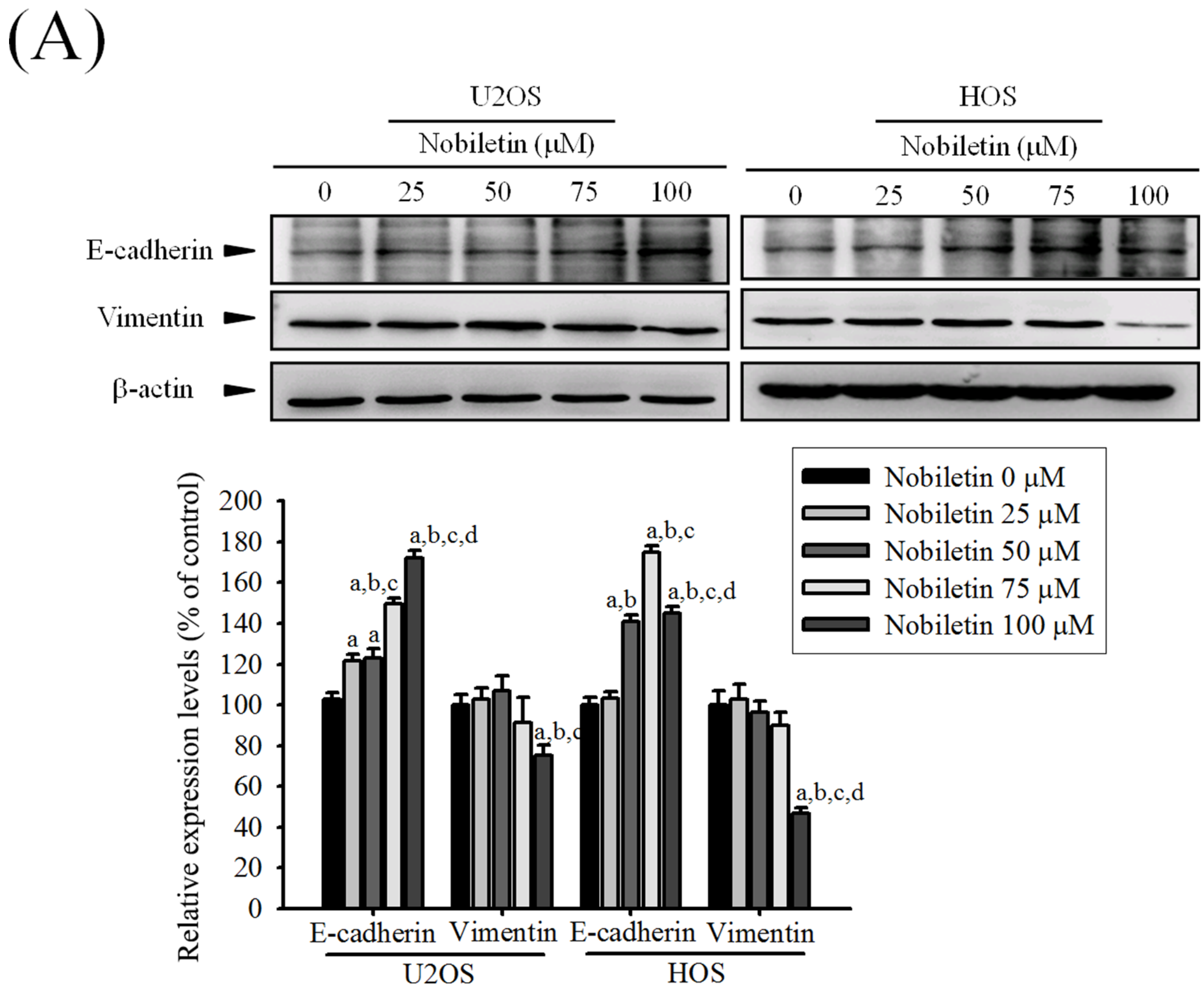

Supplementary Figure S1: Effects of nobiletin on the cell epithelial–mesenchymal transition in U2OS and HOS cells. U2OS and HOS cells were treated with nobiletin (0–100 μM) for 24 h and then subjected to western blotting to analyze the expression of E-cadherin and Vimentin. Quantitative results of E-cadherin and Vimentin protein levels, which were adjusted with β-actin protein level. Concentration effects: E-cadherin (U2OS: $F = 54.279$, $p < 0.001$; HOS: $F = 83.261$, $p < 0.001$); Vimentin (U2OS: $F = 8.049$, $p = 0.004$; HOS: $F = 14.702$, $p < 0.001$). [a]Significantly different, $p < 0.05$, when compared with 0 μM. [b]Significantly different, $p < 0.05$, when compared with 25 μM. [c]Significantly different, $p < 0.05$, when compared with 50 μM. [d]Significantly different, $p < 0.05$, when compared with 75 μM.

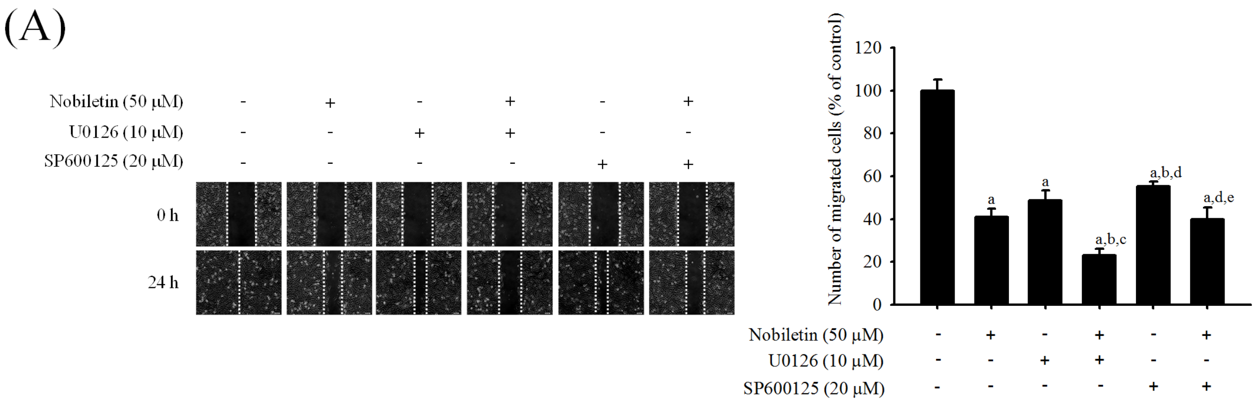

Supplementary Figure S2: Effect of nobiletin, U0126 and SP600125 on *in vitro* wound closure in U2OS cell. U2OS cells were co-treated with specific protein inhibitor U0126 or Sp600125, which incubated in the presence or absence of nobiletin (50 µM) for 24 h in a serum-containing medium. At 0 h and 24 h, phase-contrast pictures of the wounds at four different locations were taken. Cells migrating into the wound area were counted using the dash line as time zero. F =112.573, $p < 0.001$. [a]Significantly different, $p < 0.05$, when compared with 0 µM. [b]Significantly different, $p < 0.05$, when compared with noibletin-treated group. [c]Significantly different, $p < 0.05$, when compared with U0126 treated-group. [d]Significantly different, $p < 0.05$, when compared with nobiletin plus U0126-treated group. [e]Significantly different, $p < 0.05$, when compared with SP600125 treated-group.
